# Probing potential priming: Defining, quantifying, and testing the causal priming effect using the potential outcomes framework

**DOI:** 10.3389/fpsyg.2022.724498

**Published:** 2022-11-08

**Authors:** Oliver Y. Chén, Huy Phan, Hengyi Cao, Tianchen Qian, Guy Nagels, Maarten de Vos

**Affiliations:** ^1^Faculty of Social Sciences and Law, University of Bristol, Bristol, United Kingdom; ^2^School of Electronic Engineering and Computer Science, Queen Mary University of London, London, United Kingdom; ^3^The Alan Turing Institute, London, United Kingdom; ^4^Department of Psychology, Yale University, New Haven, CT, United States; ^5^Center for Psychiatric Neuroscience, Feinstein Institutes for Medical Research, Manhasset, NY, United States; ^6^Division of Psychiatry Research, Zucker Hillside Hospital, Glen Oaks, NY, United States; ^7^Donald Bren School of Information and Computer Sciences, University of California, Irvine, Irvine, CA, United States; ^8^Department of Neurology, Universitair Ziekenhuis Brussel, Jette, Belgium; ^9^Institute of Biomedical Engineering, University of Oxford, Oxford, United Kingdom; ^10^Faculty of Engineering Science, KU Leuven, Leuven, Belgium; ^11^Faculty of Medicine, KU Leuven, Leuven, Belgium; ^12^KU Leuven Institute for Artificial Intelligence, Leuven, Belgium

**Keywords:** priming effect, causal inference, potential outcomes framework, word fragment completion test, significant test, between-subjects study

## Abstract

Having previously seen an item helps uncover the item another time, given a perceptual or cognitive cue. Oftentimes, however, it may be difficult to quantify or test the existence and size of a perceptual or cognitive effect, in general, and a priming effect, in particular. This is because to examine the existence of and quantify the effect, one needs to compare two outcomes: the outcome had one previously seen the item vs. the outcome had one not seen the item. But only one of the two outcomes is observable. Here, we argue that the potential outcomes framework is useful to define, quantify, and test the causal priming effect. To demonstrate its efficacy, we apply the framework to study the priming effect using data from a between-subjects study involving English word identification. In addition, we show that what has been used intuitively by experimentalists to assess the priming effect in the past has a sound mathematical foundation. Finally, we examine the links between the proposed method in studying priming and the multinomial processing tree (MPT) model, and how to extend the method to study experimental paradigms involving exclusion and inclusion instructional conditions.

## Introduction

Imagine you are asked to fill in a fraction of a word, say _aze_ _e. Suppose the target word is gazette. What would your performance be if you have seen a list of words including gazette before the game? Intuitively, seeing a list of words containing the target answer improves one’s performance. But, how could we formally test whether the improvement exists, and how could we quantify the amount of improvement?

More specifically, we call such a phenomenon where having been exposed to an item (e.g., viewing a word or an object) facilitates the subsequent recovery of the item based on a partial or reduced perceptual cue (e.g., viewing a partial word or a fragment of an object) *repetition priming* (Hayman and Tulving, 1989; Tulving and Schacter, 1990, 1992).

Neurobiologically, this (priming) effect on word identification is facilitated and carried out through activations in the brain involving memory and learning. Although the exact neural bases of priming are as of yet little known, several lines of evidence have hinted that priming is mediated by neural systems outside of the medial temporal and diencephalic regions (Tulving and Schacter, 1990), and that priming is related to changes in cortical modules that are involved in processing specific attributes of stimulus information (Squire, 1987). Neuropsychologically, posterior cortical areas in the right hemisphere seem to be associated with object identification (Warrington and Taylor, 1978); passive reading of familiar words produces selective bilateral activation in the extrastriate cortex, suggesting that visual identification (not necessarily understanding) of words has an anterior-occipital locus (Schwartz et al., 1980; Funnell, 1983; Satori and Job, 1987; Petersen et al., 1988).

Yet, despite neurobiological and neuropsychological advances, little do we know about how to formally test the existence of a priming effect, whether the effect is causal and if so, how to quantify it. The difficulty, in part, lies in the need to compare two scenarios where only one is observable. Specifically, to claim that there exists a priming effect (e.g., the effect of a word study on word identification), one must first quantify the outcomes of two scenarios (e.g., word identification accuracy after viewing the target words vs. the accuracy without viewing the target words) and then compare these two outcomes to draw a (statistical) conclusion. But, only one of these^1^ is observable on each individual. How, then, could we compare an observable outcome with an unobservable one?

Here, linking Neyman and Rubin’s works on causal inference and Tulving and Schacter’s earlier works on priming, we aim to define, test, and quantify the causal priming effect using the *potential outcomes* framework (Neyman, 1923; Rubin, 1974, 1977, 1978). We demonstrate how to use this framework to study the priming effect by analyzing data from a between-subjects study (Hayman and Tulving, 1989). We also show that what has been previously used intuitively to study the priming effect has a sound mathematical foundation. But before we proceed, it is perhaps useful to discuss the reasons for choosing this framework, the relationship between priming and memory, and the convenience of studying priming using a word fragment completion test.

### A brief introduction of causal inference

Let us begin by briefly introducing and comparing three useful approaches to study causation: Campbell’s (“validity testing”) framework, Pearl’s (causal diagram) framework, and the Neyman–Rubin’s (potential outcomes) framework.

Campbell’s framework focuses on evaluating the validity of standard designs for experimentation in the social sciences and finding extraneous variables that may confound causal interpretations (Campbell, 1957).

The Neyman–Rubin framework focuses on the *magnitude* of the causal effect; it emphasizes the mathematical argument that can yield an analytical estimate of the causal effect. As only one of the two outcomes in the Neyman–Rubin framework can be observed from each individual, they are usually referred to as *potential outcomes* (Neyman, 1923; Rubin, 1974, 1977, 1978).

Pearl’s framework introduces *directed* graphs into causal analysis, with nodes indicating variables (e.g., exposure and outcome) and edges indicating causal links (Pearl, 1993, 1995, 2001, 2009a). In addition, the *do*(⋅) operator^2^ and the back-door and front-door criteria make some otherwise difficult causal effects identifiable (see later).

#### A comparison between Campbell’s, Neyman–Rubin’s, and Pearl’s causal models

##### Similarities

Most psychologists are familiar with Campbell’s method; perhaps few have had exposure to the Neyman–Rubin model (West and Thoemmes, 2010). In our view, however, Design 6 ^3^ in Campbell (1957) shows spirit of both Neyman–Rubin’s potential outcomes framework^4^ and Pearl’s causal diagram^5^. As for Pearl’s and Neyman–Rubin models, oftentimes, they are mathematically equivalent^6^ (see Section 7.4.4 of Pearl, 2009b).

##### Differences

Compared with Campbell’s approach, the Neyman–Rubin framework offers an analytical language for identifying and quantifying the causal effect. Compared with Neyman–Rubin formulations, Pearl’s method is oftentimes easier for social scientists to understand and visualize the causal problems using vivid graphic representations. In certain cases^7^, controlling for covariates using the Neyman–Rubin method may fail to identify a causal effect – a major criticism from the Pearl school. Furthermore, under the potential outcomes framework, there is a subtle difference between Neyman’s null (where the null hypothesis considers zero average causal effect) and Fisher’s null (where the null hypothesis considers zero individual causal effect) for many realistic situations, which may cause confusions (Ding, 2017). The Rubin school argues^8^ that causation, especially causation involving directed causation and dynamic causation, cannot be simply explained by graphs. Pearl’s method assumes that the *do*(⋅) operator itself does not perturb the (causal) system, about which some may caste doubts; in addition, oftentimes this assumption cannot be tested. For experimentalists, it is sometimes impractical to apply the *do*(⋅) operator to intervene certain variables such as gender and age. Finally, in practice, it may be difficult to obtain a complete picture of the causal diagram (e.g., the directed causal map of the brain network).

Weighing pros and cons and in light of priming research, in this article, we derive the potential causal priming framework in Neyman–Rubin’s language and accompany graphs in Pearl’s style to visualize causal relationships (see the Discussion section for future directions).

**Remark 1**. We encourage interested readers to compare, in detail, the potential outcomes framework with Campbell’s framework (e.g., West and Thoemmes, 2010) and the potential outcomes framework with Pearl’s framework [e.g., Gelman’s blog post (Gelman, 2009) and Pearl’s response under the post].

**Remark 2**. There are other fine works on causal inference; we refer our readers to them for further reading (Peters, 1941; Cochran and Chambers, 1965; Hill, 1965; Goldberger, 1972; Ding et al., 2016).

### A brief discussion of memory

#### Different memory systems

Whereas the focus of the article is on priming, it is perhaps beneficial to familiarize oneself with the memory systems, in general. This is because on the one hand, priming is related to memory, and on the other hand, it is arguably independent of explicit and semantic memory (Tulving and Schacter, 1990). By stating explicit and semantic memory, one has already implied there exists some categorization of memory systems. Although we do not intend to and cannot fully examine the hypothesis regarding the number of memory systems present, a summary of a few key classifications of memory systems may help the readers to deal with priming conceptually. Tulving (1985) argued that there exist three types of memory systems: episodic (associated with self-knowing consciousness), semantic (associated with knowing consciousness), and procedural (associated with non-knowing consciousness). Cohen and Squire (1980) and Mishkin et al. (1984) argued that there are two types of memory systems: the former coined the two systems according to the concepts of “knowing how” and “knowing that,” and the latter distinguished the habit system from the “memory” system. Others have proposed more specific classifications, arranged either hierarchically (Pribram, 1984)^9^ or interactively without a fixed relationship to each other (Johnson, 1983). More specifically to priming, it is hypothesized that there exists a pre-semantic perceptual system [called the *perceptual representation system* (PRS)] that manages priming; the PRS operates independently of the explicit and semantic memory (Tulving and Schacter, 1990). In brief, the hypothesis of the PRS suggests that there is a dissociation between priming and explicit memory and that there is a dissociation between (pre-semantic) priming and semantic memory (Warrington and Taylor, 1978; Parker et al., 1983; Graf et al., 1984; Hashtroudi et al., 1984; Cermak et al., 1985; Light et al., 1986; Shimamura, 1986; Nissen et al., 1987; Kopelman and Corn, 1988; Parkin and Streete, 1988; Tulving and Schacter, 1990).

#### Process dissociation model

Interposed between the classification of multiple memory systems and the study of priming is the need to separate the latter from other, for example, semantic and explicit memory processes. This need is partly sprawled empirically from findings where patients with amnesia reported significantly worse explicit memory (intentional use of memory) than normal subjects but showed as large a priming effect (an arguably automatic, passive use of memory) as the normal subjects (Warrington and Weiskrantz, 1974; Graf et al., 1984; Cermak et al., 1985; see Shimamura, 1986 for a review). Practically, to separate and estimate the contribution of unconscious, automatic, controlled, and intentional processes, Jacoby (1991) proposed the *process dissociation framework* and argued its utility in studying perception, memory, and thought. The key point of the framework is to use regression models to separate the effect of (consciously controlled) recollection from that of (automatic) familiarity [see Experiment 3 in Jacoby (1991) for details].

#### The role of memory in encoding instructions

Participants in a priming study need to follow instructions. Working memory, the ability to maintain and process information (Baddeley and Hitch, 1974), plays an important role in encoding both spoken (Baddeley et al., 1984; Hanley and Broadbent, 1987) and written (Wright, 1978; Wright and Wilcox, 1978) instructions. Cognitive load (including intrinsic, extraneous, and germane loads) that connects instructional design to cognitive functions is related to working memory. The cognitive load consumes a part of the working memory, and particularly, with appropriate instructional design, the germane load positively affects learning (Cooper et al., 2001).

### A brief introduction to the word fragment completion (WFC) test

**FIGURE 1:**
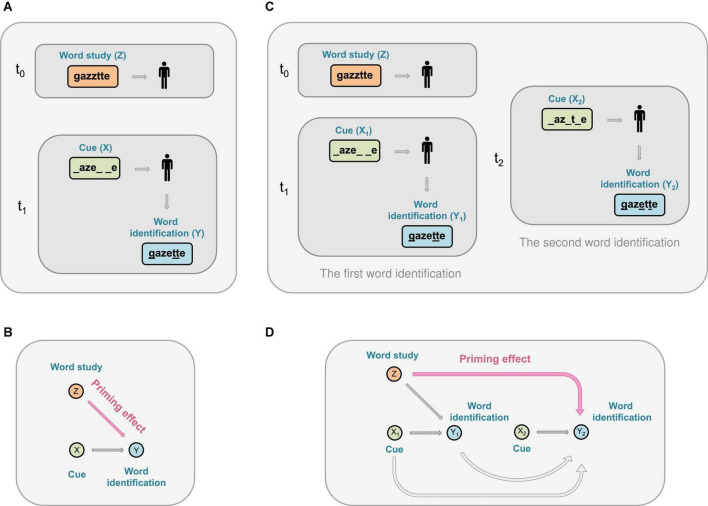
A schematic representation of the priming effect during a word completion experiment. **(A)** First, a subject views a list of words (including a target word gazette, or Z) during a word study. Next, a cue that consists of a fragment of the word (e.g., _aze_ _e, or X) is given to the subject. The subject is then asked to fill in the blanks. The subject may successfully uncover the target word or another word gazelle; or report a non-word gazeele, or an incomplete entry such as _azelle. **(B)** Priming effect refers to the phenomenon that being previously exposed to the word gazette (Z) primes (i.e., facilitates) the identification of the word (Y) given a partial or reduced cue (X). **(C)** A word study followed by two-word identifications. Left: The same word study and the subsequent cue-based identification, as in **(A)**. Right: An additional cue-based identification. In the figure, a different cue (e.g., _az_t_e, or X_2_) is used during the second word identification for illustration purposes; two identical cues can also be used. **(D)** Priming effect where being previously exposed to the word gazette (Z) primes (i.e., facilitates) the identification of a word (Y_2_) during a second word identification test given a partial or reduced cue _az_t_e (X_2_).

The word fragment completion (WFC) test is widely used to assess priming. In general, the test consists of a study phase and a test phase. During the study phase, subjects are instructed to view a list of words, including target words (e.g., gazette) and non-target words (called buffers). The test phase starts after an interval (e.g., 2 h). During this phase, the subjects are randomly assigned into two groups; each group undertakes one of the following tasks: (1) uncovering studied words (e.g., gazette) given a cue and (2) uncovering non-studied words given a cue. Some fragment completion tests will include an additional test, which involves repeating the word identification of gazette either with the same cue or with a different cue (see examples in the Results section). In simple terms, priming is said to have occurred when the success rate of cue-based item identification after studying the item is higher than that of a non-studied item (see Figure 1).

Under the Neyman–Rubin’s potential outcomes framework, the priming effect of receiving a word study, which consists of the target words (the exposure of interest), on word identification (the outcome) can be defined as follows:

*It is the difference between the two potential outcomes: the first is the outcome had an individual received the word study which consists of the target words, and the second is the outcome had the same individual not received the word study (or received a word study which did not contain the target words).*

We restrict our focus on the priming effect during a non-semantic word completion test, although the framework can be extended to studying semantic tasks such as rating the pleasantness when viewing a word and giving its definition. This is, in part, because priming is not affected by semantic and non-semantic encoding (Tulving and Schacter, 1990). Similarly, as priming occurs in more complex studies such as visual object recognition (Schacter et al., 1990), the framework introduced in this article may also be useful to quantify these priming effects. Although we focus on modeling the causal effect in studies of implicit memory, it may shed some light on studies of explicit memory (see the Discussion section). Finally, we note that when the instructions were not implicit but explicit during a fragment completion test, the test should be, in spirit, considered more as a “cued recall test” than a “fragment completion test.”

## Method

### Notations and definitions

We begin by defining the notations used throughout this article. We use *Z* to denote whether a word study concerning viewing a list of words (including target words, such as gazette, which we use as an example throughout this article, and non-target words, such as *vermouth*) is undertaken at time *t*_0_ = 0 (see Figure 1). Specifically, *Z* = 1 means that a subject has undertaken a word study including the target words (and henceforth referred to as having undertaken a word study for simplicity), and *Z* = 0 means that the subject has not undertaken the word study (or have undertaken a word study with all non-target words, which, for simplicity, we will henceforth refer to as not having undertaken a word study). In this study, we consider that *Z* takes binary values (i.e., having vs. not having conducted a word study), although our approach can be extended to categorical *Z* that takes more than two values (e.g., word studies consisting of words with low, intermediate, and advanced level of complexity). The word complexity can be quantified by, for example, evaluating the combination of syllable shapes and word patterns. As such, a further extension of *Z* can take any value between 0 and 100 to indicate complexity of each word (see the Discussion section for continuous and time-dependent cases).

Let *X*_1_ denote a cue (e.g., *X*_1_ = _aze_ _e) given during a WFC test at a time *t*_1_ (*t*_1_ = *t*_0_, typically *t*_1_ is 2 h after *t*_0_) (see Figure 1). We write Y(*X* = x, *Z* = z) as the outcome of word identification on the experiment unit (i.e., an individual participant), given that the unit received a word study *Z* = *z* at *t*_0_ and a cue *X* = *x*, where the upper case indicates a random variable and the lower case refers to its realized value. If there is an additional test, let *X*_2_ denote the cue (e.g., *X*_2_ = _az_t_e) given during the second word completion test at time *t*_2_, where *t*_2_ can be, for example, 2 h after *t*_1_. By design, we have *t*_2_ > *t*_1_ > *t*_0_. Between *t*_1_ and *t*_2_, participants can undertake tasks irrelevant to the experiment, such as taking a cognitive psychology class. We define *Y*_1_ and *Y*_2_ as the corresponding word identification outcomes given cues *X*_1_ and *X*_2_, respectively.

In the following, we always assume that each cue X corresponds to a single answer, and we drop the notational dependence of Y on the target word where there is no confusion. For example, Y(*X* = _*aze*__e, *Z* = 1) = 1 means that the word identification is correct (e.g., the identified word is gazette, the target word, or gazelle, another correct answer^10^) given the cue *X* = _aze_ _e, after a word study *Z* consisting of a target word gazette. Y(*X* = _*aze*__e, *Z* = 1) = 0 means that the word identification is incorrect given the cue *X* = _aze_ _e, after a word study *Z* consisting of the target word gazette. Similarly, Y(*X* = _*aze*__e, *Z* = 0) = 1 means that the word identification is correct (i.e., the identified word is gazette or gazelle) given the cue *X* = _aze_ _e, had a word study *Z* not been conducted. Y(*X* = _*aze*__e, *Z* = 0) = 0 means that the word identification is incorrect given the cue *X* = _aze_ _e, had a word study *Z* not been conducted.

#### Definition 1.1 (Causal priming effect)

The causal priming effect on an experiment unit (i.e., a subject) given a cue *X* = x is defined as Y (*X* = x, *Z* = 1)−Y(*X* = x, *Z* = 0); this quantifies the difference between the outcome *Y* from a study unit that has conducted a word study (*Z* = 1) versus the outcome *Y* from the same unit had no word study been conducted (*Z* = 0), given the same cue *X* = x.

**FIGURE 2:**
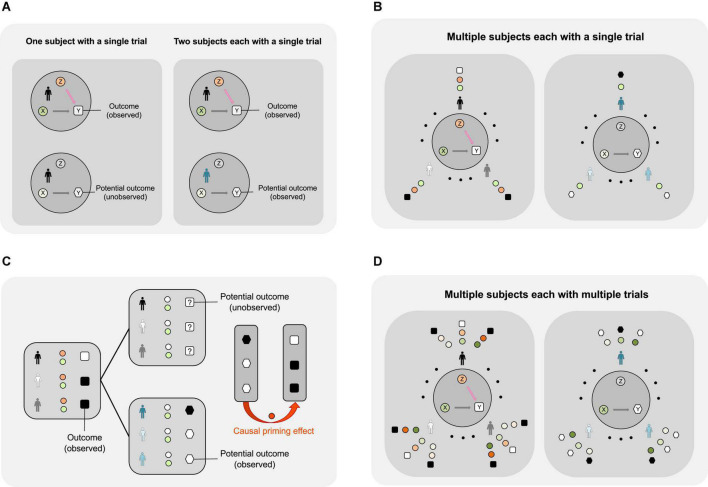
Using the potential outcomes framework to study the causal priming effect during a word completion experiment. **(A)** Identifying the causal priming effect with one trial. Top left: The figure describes a scenario where the subject first conducts a word study (*Z* = 1) and then aims to identify a target word based on a partial cue (X). The outcome (or Y) is observable. Bottom left: The figure describes a scenario where a subject does not conduct a word study (*Z* = 0) and aims to identify a target word based on a partial cue (X). If the subject has already participated in a word study, this (potential) outcome (or Y) under no word study is not observable. Top right: The same experiment as in the top left. *Bottom right*: Since the potential outcome of the subject (indicated by a human icon in black) is not observable, a different subject (indicated by a human icon in blue) is asked to identify the same target word based on the same partial cue (X), without a word study (*Z* = 0). If the two subjects are similar, then the causal priming effect is estimated by the difference between the outcomes (denoted by a letter Y in a square and a letter Y in a hexagon). **(B)** To reduce the possibility that a particular word may yield various priming effects on different subjects, multiple individuals are randomized to either conduct a word study (*Z* = 1) or not (*Z* = 0). The individuals with grayscale colors receive a word study; the individuals with bright colors do not receive a word study. For the left image, green circles represent various cues, orange circles represent word studies (*Z* = 1), and black or white squares represent the word study outcomes, where a black square indicates success (*Y* = 1) and a white square indicates failure (*Y* = 0). For the right image, green circles represent the cues corresponding to those on the left image, gray circles with the letter Z refer to having not conducted a word study (*Z* = 0), and black or white hexagons represent the word study outcomes, where a black hexagon indicates success (*Y* = 1) and a white hexagon indicates failure (*Y* = 0). **(C)** Average priming effect of a word study on word identification among multiple subjects using one trial. Left: Half of the subjects are randomized to perform the word identification experiments after a word study (indicated by orange circles). The black and white squares indicate the observed outcomes. Top middle: The potential outcomes of the same subjects (who have participated in a word study) had they not participated in a word study (indicated by blank circles). The squares with question marks indicate that these outcomes are not observable. Bottom middle: The remaining subjects perform the word identification experiments without a word study; the outcomes are observable and are indicated by black and white hexagons for successful and failed cases, respectively. Right: Due to randomization, the difference in the outcomes between the two groups (divided by the sample size) gives an estimate of the average priming effect of the word study on word identification in a sample. **(D)** The experiment can be further extended to multiple subjects with multiple words.

Only one of the two potential outcomes can be observed from each subject. In other words, the individual-level causal priming effect is non-identifiable. Therefore, a natural inquiry into the causal priming effect is to uncover the average priming effect across multiple subjects (see Figures 2A,B).

#### Definition 1.2 (Average priming effect of a single studied word)

Consider one target word in a wordlist that is viewed by a total of *N* subjects. Then, the *average priming effect* (APE) of a word study *Z* on a word identification Y given a cue X is defined as follows:


$$APE_{N}^{\left\{1\right\}}=\frac{1}{N}\left\{\sum_{i=1}^{N}Y_{i}\left(X=x,Z=1\right)-Y_{i}\left(X=x,Z=0\right)\right\}$$

where Y_*i*_(*X* = x, *Z* = z) indicates the word identification result of the *i^th^* subject after given a cue *X* = *x* and a word study *Z* (*Z* = 1 means after viewing a wordlist consisting of target words and *Z* = 0 means without a word study). The superscript {1} indicates that it is the average priming effect for one target word, and the subscript *N* indicates that the effect is defined on *N* individuals.

Unfortunately, we cannot observe both Y (*X* = x, *Z* = 1) and Y(*X* = x, *Z* = 0) on the same subject. This is because after having assigned (or not assigned) a word study *Z* (e.g., *Z* = 1) and the word identification test result Y has been reported, we cannot go back to the time *t*_0_ to assign a different *Z* (i.e., *Z* = 0). Certainly, one could experiment on the same unit in two trials (one with the word study *Z* = 1 and the other with *Z* = 0), which consists of a repeated-measures study (e.g., Challis and Brodbeck, 1992). The first study, however, may have a carryover or learning effect on the second. Therefore, we cannot ascertain that the priming effect is due to the word study *Z* or the information learned (e.g., the cue *X*, the word identification *Y*, or the study mechanism) from the first test (see the Discussion section for details).

### Estimating average priming effect involving one target word in a *2K* trial study

Consider a sample of *2K* subjects, where half of the subjects undertake a word study and half do not (see Figure 2B). Let *S_Z_* denote the indices of the subjects who undertake the word study, and let *S*_*NZ*_ denote the indices of the subjects who do not. Let *DPE* denote the difference between the average observed word identification accuracy of the *S_Z_* group and the average observed word identification accuracy of the *S*_*NZ*_ group, as follows:


$$DPE_{2K}^{\left\{1\right\}}=\frac{1}{K}\left\{\sum_{i\in S_{Z}}{\text{Y}}_{i}\left(\text{X}=x,Z=1\right)\right\}-\frac{1}{K}\left\{\sum_{i\in S_{NZ}}{\text{Y}}_{i}\left(\text{X}=x,Z=0\right)\right\}.$$

Following the definition of the *APE* in *Definition 1.2*, the average (causal) priming effect across a sample of *2K* individuals involving one target word is defined as follows (see Figure 2C):


$$APE_{2K}^{\left\{1\right\}}=\frac{1}{2K}\left\{\sum_{i=1}^{2K}{\text{Y}}_{i}\left(\text{X}=x,Z=1\right)-{\text{Y}}_{i}\left(X=x,Z=0\right)\right\}.$$

Since $APE_{2K}^{\left\{1\right\}}$ is not observable and $DPE_{2K}^{\left\{1\right\}}$ is, one would ask if $DPE_{2K}^{\left\{1\right\}}$ is close to $APE_{2K}^{\left\{1\right\}}$. The answer depends on two factors: (a) how well matched are subjects who conduct the word study and those who do not; (b) if the word study is randomly assigned. We examine these factors in detail as follows:

First, if the *S_Z_* group and the *S*_*NZ*_ group are perfectly matched^11^ [i.e., for every subject in the *S_Z_* group who receives a word study, there is a subject in the *S*_*NZ*_ group who does not receive a word study; and these two (matched) subjects would perform identically if a word study were conducted or if a word study were not conducted^12^], then $DPE_{2K}^{\left\{1\right\}}=APE_{2K}^{\left\{1\right\}}$. This holds whether the word study *Z* is randomly assigned or not (Rubin, 1974). Second, if the two groups are not perfectly matched, but before the tests, investigators have controlled all the variables that would affect the performance (e.g., only consider subjects with the same age, gender, and education background), then $DPE_{2K}^{\left\{1\right\}}$ is close to $APE_{2K}^{\left\{1\right\}}$ (i.e., the subjects are as if matched). Third, if the word study *Z* is randomly assigned, even if there are unmatched subjects (e.g., subjects have significant different language proficiency). For example, English speakers may perform better than non-English speakers in a word completion test in English; the random assignment is going to balance, in expectation, all observed and unobserved factors that would impact the word identification. To put it more concretely, by randomly assigning a word study to individuals, some English speakers would receive a word study (the rest of the English speakers would not receive one), and some non-English speakers would receive a word study (the rest non-English speakers would not receive one). As a result, the individuals who receive a word test consist of both English and non-English speakers, and the individuals who do not receive a word test also consist of both English and non-English speakers; thus, the bias due to language efficiency is reduced. Randomization becomes increasingly effective when the sample size *N* increases (Scheffe, 1959; Rubin, 1974; Wu and Hamada, 2000; Hinkelmann and Kempthorne, 2005).

Although matching or randomization makes $DPE_{2K}^{\left\{1\right\}}$ a suitable estimator for $APE_{2K}^{\left\{1\right\}}$, it remains important to generalize it to any *2K* sample. To that end, we defined the expected priming effect (EPE) (i.e., the expectation of $DPE_{2K}^{\left\{1\right\}}$) as follows:


$$EPE_{2K}^{\left\{1\right\}}=𝔼\left\{\frac{1}{K}\sum_{i\in S_{Z}}I_{{\text{Y}}_{i}\left(\text{X}=x,Z=1\right)=1}-\frac{1}{K}\sum_{i\in S_{NZ}}I_{{\text{Y}}_{i}\left(\text{X}=x,Z=0\right)=1}\right\}$$

where 𝔼 indicates the expectation operation.

Since Y_*i*_ = 0 or 1, then $DPE_{2K}^{\left\{1\right\}}=\frac{1}{K}\sum_{i\in S_{Z}}$ $I_{{\text{Y}}_{i}\left(\text{X}=x,Z=1\right)=1}-\frac{1}{K}\sum_{i\in S_{NZ}}I_{{\text{Y}}_{i}\left(\text{X}=x,Z=0\right)=1}$, where *I*_Y_*i*__(X = x, Z = z) = 1 is an indicator function^13^ that takes value 1 if Y_*i*_(X = x, Z = z) = 1, and takes value 0 if Y_*i*_(X = x, Z = z) = 0. Then $EPE_{2K}^{\left\{1\right\}}$ reduces to


(1) $$EPE_{2K}^{\left\{1\right\}}=\mathbb{P}\left\{\text{Y}\left(\text{X}=x,Z=1\right)=1\right\}-\mathbb{P}\left\{\text{Y}\left(\text{X}=x,Z=0\right)=1\right\}$$

where ℙ{Y (X = x, Z = z) = y} denotes the probability of the word identification *Y* equals to y (y = 0 or *1*) given the cue *X* = x and the word study *Z* equals to *z* (*z* = 0 or *1*).

In simple terms (see Remark 3), $EPE_{2K}^{\left\{1\right\}}$ means that the expected priming effect estimated from *2K* subjects regarding one word is the difference between the probability of correctly identifying the target word for all subjects who have taken the word study (*Z* = 1) and the probability of correctly identifying the target word for those who have not participated in the word study (*Z* = 0).

### Estimating priming effect involving multiple target words

The variability of individual memory affects the individual priming effect (a treatment of which is to estimate the average priming effect across subjects, as outlined in *Definition 1.2*) and so does the variability of words. Hence, the *APE*estimated using a complicated, uncommon, and non-word is likely to differ from the *APE* estimated using a simple and common word; this is true even when words of similar complexity are considered (because even when we only focus on, say, words of intermediate complexity, there are, potentially, differences in syllable shapes and word patterns). A natural treatment is to conduct tests on multiple words and estimate the average priming effect over these words across subjects.

#### Definition 1.3 (Average priming effect across multiple studied words)

Consider a wordlist consisting of *M*target words in a study consisting of a total of *N* subjects (see Figure 2D). Define X_*ij*_ as a cue given to the *i^th^* subject associated with the *j^th^* target word. Define Y_*ij*_ as the outcome of the corresponding word identification. Then, the *average priming effect* of the word study *Z* across *M* words on multiple word identifications is defined as follows:


$$APE_{N}^{\left\{M\right\}}=\frac{1}{NM}\left\{\sum_{i=1}^{N}\sum_{j=1}^{M}{\text{Y}}_{ij}\left({\text{X}}_{ij}={\text{x}}_{ij},Z=1\right)-{\text{Y}}_{ij}\left({\text{X}}_{ij}={\text{x}}_{ij},Z=0\right)\right\}$$

where Y_*ij*_ (X_*ij*_ = x_*ij*_, Z = z) indicates the word identification result of the *j^th^* target word from the *i^th^* subject after given the cue X_*ij*_ = x_*ij*_ and the word study *Z* (*Z* = 1 means after viewing a wordlist consisting of target words and *Z* = 0 means without the word study). The superscript {*M*} indicates that it is the average priming effect for *M* (*M* ≥ 2) target words, and the subscript *N* indicates that the estimate is obtained from a sample of *N* subjects.

Again, $APE_{N}^{\left\{M\right\}}$ is not observable. The observable *DPE* in a study consisting of *N* = 2*K* subjects and *M* target words (between the group given a word study and the group not given a word study) is as follows:


(2) $$DPE_{2K}^{\left\{M\right\}}=\frac{1}{KM}\left\{\sum_{i\in S_{Z}}\sum_{j=1}^{M}{\text{Y}}_{ij}\left({\text{X}}_{ij}={\text{x}}_{ij},Z=1\right)\right\}$$

$$\;-\frac{1}{KM}\left\{\sum_{i\in S_{NZ}}\sum_{j=1}^{M}{\text{Y}}_{ij}\left({\text{X}}_{ij}={\text{x}}_{ij},Z=0\right)\right\}.$$

Similar to a *2K* trial study concerning one target word, the expected priming effect reduces to


(3) $$EPE_{2K}^{\left\{M\right\}}=\frac{1}{M}\sum_{j=1}^{M}EPE_{2K,j}^{\left\{1\right\}}$$

where $EPE_{2K,j}^{\left\{1\right\}}$ refers to $EPE_{2K}^{\left\{1\right\}}$ for the *j^th^* word.

In simple terms, $EPE_{2K}^{\left\{M\right\}}$ means that the expected priming effect estimated from *2K* subjects across *M* words is the difference between the probability of corrected identifying *each* of the *M* target words for all subjects who have participated in the word study (*Z* = 1) and the probability of correctly identifying the corresponding word for all subjects who have not participated in the word study (*Z* = 0) averaged over *M* words. For simplicity, let us denote ℙ{Y (X = x, Z = 1) = 1} as *p*_1_ and ℙ{Y (X = x, Z = 0) = 1} as *p*_0_, which can be estimated by $\frac{1}{KM}\left\{\sum_{i\in S_{Z}}\sum_{j=1}^{M}{\text{Y}}_{ij}\left({\text{X}}_{ij}={\text{x}}_{ij},Z=1\right)\right\}$ and $\frac{1}{KM}\left\{\sum_{i\in S_{NZ}}\sum_{j=1}^{M}{\text{Y}}_{ij}\left({\text{X}}_{ij}={\text{x}}_{ij},Z=0\right)\right\}$, respectively.

**Remark 3. Eqs.** (1, 3) are analytical solutions to estimating the priming effect for one target word and *M* target words, respectively. They have been used intuitively by experimentalists; the aforementioned arguments demonstrate the mathematical validity of such usages in practice.

### Connecting the potential outcomes framework with multinomial processing tree (MPT) models in studying priming

**FIGURE 3:**
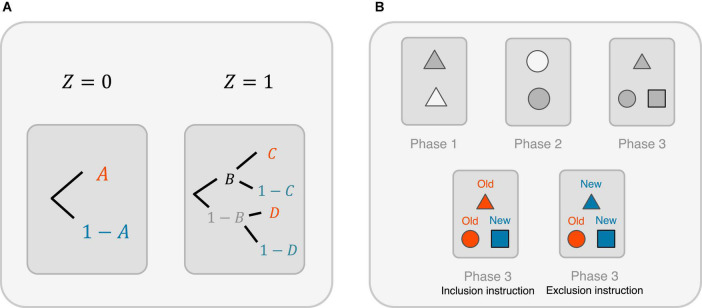
Linking the potential outcomes-based priming research with two prominent quantitative psychological methods. **(A)** Multinomial processing tree (MPT) model for studying the priming effect. Here, *A* and 1 − *A* are the probabilities of correctly and incorrectly, respectively, identifying the words without a word study (i.e., given *Z* = 0); *B* and 1 − *B* are the probabilities of storing (consciously or unconsciously) and not storing, respectively, the studied word after a word study (i.e., given *Z* = 1); *C* and 1 − *C* are the probabilities of correctly and incorrectly, respectively, identifying the words if the studied words are stored in the memory; *D* and 1 − *D* are the probabilities of correctly and incorrectly, respectively, identifying the words if the studied words are not stored in the memory. **(B)** A three-phase experiment with exclusion and inclusion instructional conditions. Top left: A set of items is shown during the Phase 1 study. Top middle: Another set of items (different from those in Phase 1) is shown during Phase 2. Top right: During Phase 3, the participants are given a list of items consisting of those who have appeared during Phases 1 and 2 and distractor items that have not appeared before. Subsequently, they are asked to classify them into either “old” or “new” following an inclusion instruction or an exclusion instruction. Bottom left: Under the inclusion instruction, participants need to call an item old if it has appeared in either Phase 1 or 2 and call a distractor item new. Bottom right: Under the exclusion instruction, participants need to call an item old only if it has appeared in Phase 2, and new otherwise.

It turns out that the potential outcome framework-based priming study discussed here can be linked to the priming study using the multinomial processing tree (MPT) model (Batchelder and Riefer, 1999; Erdfelder et al., 2009). To see this, consider a word fragment completion test using an MPT diagram (see Figure 3A).

Here, let us denote *A* and 1 − *A* as the probabilities of correctly and incorrectly, respectively, identifying the words without a word study (i.e., given *Z* = 0). Let us denote *B* and 1 − *B* as the probabilities of storing (consciously or unconsciously) and not storing, respectively, the studied word after a word study (i.e., given *Z* = 1). Let *C* and 1 − *C* be the probabilities of correctly and incorrectly, respectively, identifying the words if the studied words are stored in the memory; let *D* and 1 − *D* be the probabilities of correctly and incorrectly, respectively, identifying the words if the studied words are not stored in the memory.

Naturally, *p*_1_ = *BC* + *D*(1 − *B*) and *p*_0_ = *A*, where *p*_1_ and *p*_0_ are defined previously, and the priming effect estimated using the potential outcomes framework is *p_1_ − p*_0_. We have *p*_1_ − *p*_0_ = {*BC* + *D*(1 − *B*)} − *A* = *B*(*C* − *D*) + (*D* − *A*). Note that (1) *B*(*C* − *D*) is the product of consciously or unconsciously storing information from the word study into the memory (i.e., *B*) and the improvement^14^ of word identification accuracy, thanks to the stored information [i.e., (*C* − *D*)]; (2) (*D* − *A*) gives the difference between the probability of correctly uncovering words after a word study, even though no information from the word study has been added into the memory (to correctly identify words, one, therefore, has to either actively retrieve existing knowledge or use guessing), and the probability of uncovering words without a word study (which also relies on either existing knowledge or guessing).

Furthermore, it is not unfair to assume that *A* equals or is very close to *D*. Suppose *A* = *D*, then the relationship between the priming effect identified using the potential outcomes framework and the MPT model simplifies to *p*_1_ − *p*_0_ = *B*(*C* − *D*). In other words, the potential priming effect (i.e., *p_1_ − p*_0_) chiefly depends on two factors: first, the consciously or unconsciously stored memory from the word study (i.e., *B*); second, the improvement of word identification accuracy, thanks to the stored information (i.e., (*C* − *D*)).

The aforementioned argument can be extended to studying multiple, successive word identification phases. We leave this to our readers as an exercise.

### Extending the framework to experimental paradigms with exclusion and inclusion instructional conditions

Consider a three-phase study (see Figure 3B), where two sets of different items are shown during Phases 1 and 2 for learning purposes, and during Phase 3, the participants are given a list of items consisting of items that have appeared during Phases 1 and 2 and distractor items that have not appeared before. Subsequently, they are asked to classify them into either “old” or “new” following an inclusion instruction or an exclusion instruction (Buchner et al., 1995). Under an inclusion instruction, the participants need to call an item *old* if it has appeared in either Phase 1 or 2 and call a distractor item *new*. Under an exclusion instruction, the participants need to call an item *old* only if it has appeared in Phase 2, and *new* otherwise.

The framework proposed in this article can also be modified to study the experimental paradigm with exclusion and inclusion instructional conditions. To demonstrate this, let us define *Z*_1_ and *Z*_2_ as two lists of items during Phases 1 and 2, respectively. Let *X* and *X*′ denote the outcomes of the identification during Phase 3 under inclusion and exclusion instructions, where their realizations are either {new} or {old} for each given item. Following similar arguments as before, we define the potential difference between the results from the inclusion and exclusion instructions as follows:


$$\frac{1}{NM}\{\sum_{i=1}^{N}\sum_{j=1}^{M}{\text{Y}}_{ij}\left({\text{X}}_{ij}={\text{x}}_{ij},Z_{1}=1,Z_{2}=1\right)-{\text{Y}}_{ij}\left(X_{ij}^{\prime }=x_{ij}^{\prime },Z_{1}=1,Z_{2}=1\right)\}.$$

Note that here, it is assumed that the inclusion and exclusion instructions are given to the same participants in a group. This is not ideal as repeating Phase 3 under different conditions may bias the results. Using the potential outcome framework, this scenario can be estimated as follows:


$$\frac{1}{KM}\left\{\sum_{i\in S_{I}}\sum_{j=1}^{M}{\text{Y}}_{ij}\left({\text{X}}_{ij}={\text{x}}_{ij},Z_{1}=1,Z_{2}=1\right)\right\}$$

$$-\frac{1}{KM}\left\{\sum_{i\in S_{E}}\sum_{j=1}^{M}{\text{Y}}_{ij}\left(X_{ij}^{\prime }=x_{ij}^{\prime },Z_{1}=1,Z_{2}=1\right)\right\}$$

where the two parts (before and after the minus sign) are estimated from subjects in groups *S_I_* (following the inclusion instruction) and *S_E_* (following the exclusion instruction), respectively. Note that the aforementioned result equals to *p*_*i*−_*p*_*e*_ in Buchner et al. (1995), which quantifies the probability of consciously recollecting a Phase 1 item.

### Testing the significance of the priming effect

Returning to the priming study, although the focus of this article so far has been to define and quantify the causal priming effect, it may also be important for investigators to test whether a detected causal effect is significant. For example, consider 100 subjects who have undertaken the word study and 100 people who have not undertaken the word study. Suppose the estimated expected priming effect (*EPE*) is 0.1; is 0.1 in a sample of 200 subjects significant (from 0, where 0 indicates no priming effect)? What if the estimated *EPE* is 0.05?

One way to answer this question is to conduct a hypothesis test on whether the estimated priming effect is significant; that is, to verify the (alternative) hypothesis that the *EPE* is significantly greater than zero. Thanks to **Eqs.** 1, 3, the *EPE* can be written in terms of probability and can therefore be examined using a proportion test (Ott and Longnecker, 1980; Bickel and Doksum, 2000).

Formally, the test statistic is defined as follows:


(4) $$z=\frac{DPE}{\sqrt{\hat{p}\left(1-\hat{p}\right)\left(\frac{1}{N_{1}}+\frac{1}{N_{0}}\right)}}$$

where ${\hat{p}}_{1}=\frac{1}{KM}\sum_{i\in S_{Z}}\sum_{j=1}^{M}{\text{Y}}_{ij}\left({\text{X}}_{ij}={\text{x}}_{ij},Z=1\right)$, ${\hat{p}}_{0}=\frac{1}{KM}\sum_{i\in S_{NZ}}\sum_{j=1}^{M}{\text{Y}}_{ij}\left({\text{X}}_{ij}={\text{x}}_{ij},Z=0\right)$, $DPE={\hat{p}}_{1}-{\hat{p}}_{0}$, $\hat{p}=\frac{1}{2KM}\sum_{i\in S_{Z}\cup S_{NZ}}\sum_{j=1}^{M}{\text{Y}}_{ij}\left({\text{X}}_{ij}={\text{x}}_{ij},Z=\left\{0,1\right\}\right)$, and *N*_1_ = *N*_2_ = *KM* ^15^. Here, the *DPE* is the empirical estimate of the *EPE* obtained from **Eq.** 2 and $\hat{p}$ is the pooled probability (from both groups) of correct word identification (in other words, the overall probability of correctly identifying a word when the group that undertaken a word study and the group that did not undertake a word study are combined). The hat symbol, for example, in ${\hat{p}}_{1}$ is an estimate of *p*_1_.

One can then compare the *p*-value associated with the *z* score to evaluate the significance. Note that the aforementioned *z*-test is the same as a Chi-square test, where the *z*-statistic is equal to the square root of the Chi-square statistic, and the *p*-values of the two tests are identical. When the word studies are multivariate (e.g., there are more than two types of word study), continuous (e.g., the word study involves words with different degrees of complexity), or time-dependent (e.g., several tests are carried out with large time intervals in between), more advanced statistical tests can be used (see the Discussion section for details).

Subsequently, the 100 (1 − α) percent confidence interval (Wilson, 1927; Newcombe, 1998) for the estimated priming effect is as follows:


$$(DPE-z_{\left(1-\frac{\alpha }{2}\right)}\sqrt{\frac{{\hat{p}}_{1}\left(1-{\hat{p}}_{1}\right)}{N_{1}}+\frac{{\hat{p}}_{0}\left(1-{\hat{p}}_{0}\right)}{N_{0}}},\;DPE+z_{\left(1-\frac{\alpha }{2}\right)}\sqrt{\frac{{\hat{p}}_{1}\left(1-{\hat{p}}_{1}\right)}{N_{1}}+\frac{{\hat{p}}_{0}\left(1-{\hat{p}}_{0}\right)}{N_{0}}}).$$

### Estimating priming effects with covariates

Although the word study *Z* is the primary factor that affects the outcome *Y*, it remains possible that there exist additional variables (denoted as *W*) that, if not considered, may bias the estimation of the causal priming effect. These variables could either have a causal relationship with the word identification outcome *Y* (e.g., take *W* as intelligence) or are spuriously (i.e., by chance) correlated with the outcome in the sample (e.g., one’s height). Randomization only ensures that in expectation, the covariates are balanced between the two treatment groups. There, however, could still be chance imbalances in the covariates between the two treatment groups; in this case, adjusting for the covariates will increase the signal-to-noise ratio^16^ and make the priming effect more likely to be detected, if exists.

For example, take *W* as one’s IQ, which may affect word identification. Consider 20 subjects with a mean IQ of 100 (10 with IQ larger than 100 and 10 with IQ less than 100). Certainly, we could create two splits with each split containing five individuals with above-average IQ and five with below-average IQ. Our point is that sometimes, such a balanced sample is difficult to obtain, and thus, protective measures need to be taken instead (a good example here is the proficiency in English language – another variable that may affect word identification). In practice, however, it is difficult and costly for researchers to collect samples that contain subjects that are perfectly matched. Thus, we proceed here assuming such a (not completely matched) case occurs. For example, if we are to randomly assign a word study (*Z* = 1) to 10 subjects and no word study (*Z* = 0) to another 10 subjects, the group with the word study may contain eight subjects with above-average IQ and the other group with two subjects with above-average IQ. Then, the result using **Eq.** 2 could potentially over-estimate the priming effect since there are more people with above-average IQ in the word study group.

The effect of an additional variable can be adjusted in a logistic regression model. Specifically, consider


$$logit\left(\mathbb{P}\left\{{\text{Y}}_{i}\left(\text{X}=x,Z=z_{i}\right)=1\right\}\right)=\beta_{0}+{\text{β}}_{z}z_{i}+{\text{β}}_{w}w_{i}$$

where *z_i_* indicates whether the *i^th^* subject receives a word study or not, *w_i_* is the IQ for the *i^th^* subject, β_0_ is the estimated intercept, and β_*z*_ and β_*w*_ are the estimated parameters for *z_i_* and *w_i_*, respectively. The estimated β_*z*_ then indicates the priming effect from *Z*, when it is adjusted for the IQ effect (W). Specifically, controlling (i.e., removing) the effect from IQ to word identification *Y*, β_*z*_ quantifies the priming effect: the probability of correctly identifying a word increases $\frac{e^{\beta_{z}}}{1+e^{\beta_{z}}}$ when an individual conducts a word study versus not conducting a word study. Again, the logistic formula is stated for a word study considering one target word with the same cue *X* = *x* and can be relatively easily extended to a study considering multiple words and multivariate covariates.

In the following, we will perform data analysis using data from a between-subjects study (Hayman and Tulving, 1989) to demonstrate how to use the framework to study the potential causal priming effect. The advantage of using a between-subject study is that the priming effect can be evaluated when the same experiment cannot be run on the same subjects more than one time; it may also reduce the likelihood of carryover or learning effect in a repeated-measures design (see section “Discussion”).

## Results

**FIGURE 4:**
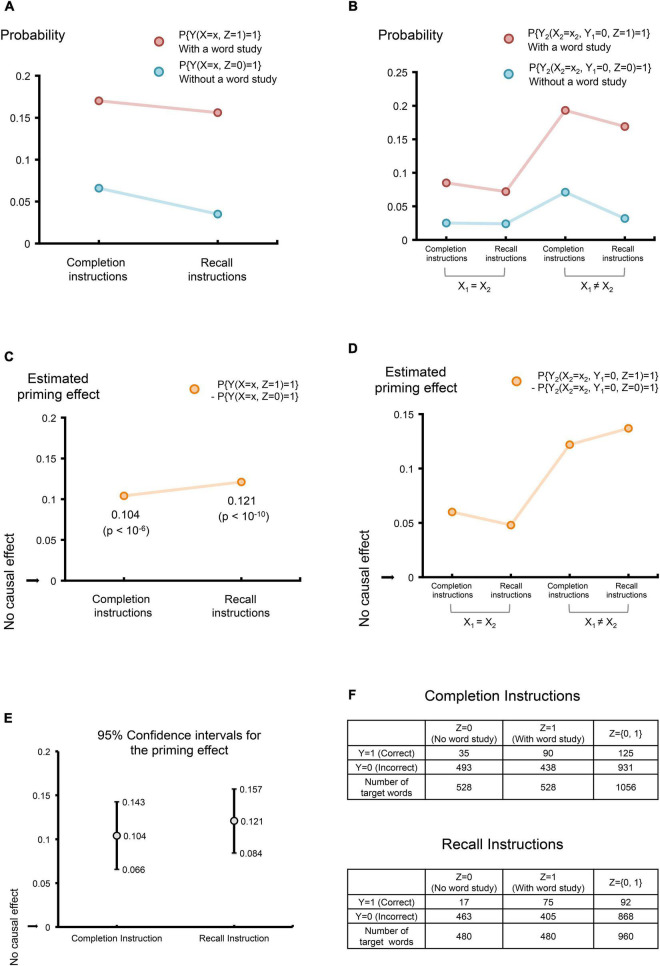
Estimating the causal priming effect. **(A)** Probabilities of correctly uncovering target words given fractional cues, under the completion instructions and the recall instructions. The color red is used to indicate experiments involving a word study including target words; the color blue is used to indicate experiments involving a word study without target words (abbreviated as “without a word study”). **(B)** The probabilities of correctly uncovering target words during a second cue-based test, given that the first test failed to uncover the same word. Four experimental sceneries were considered, combining two instruction strategies: the completion instructions and the recall instructions, and two types of cues: the same cues and different cues during two tests. *X*_1_ and *X*_2_refer to cues from the first test and the second test, respectively; *X*_1_ = *X*_2_ indicates the same cues were used in the two tests; *X*_1_ ≠ *X*_2_ indicates different cues were used in the two tests. Again, the color red is used to indicate experiments involving a word study including target words; the color blue is used to indicate experiments involving a word study without target words (abbreviated as “without a word study”). **(C)** The estimated causal priming effects and their *p*-values correspond to **(A)**. **(D)** The estimated causal priming effects correspond to **(B)**. **(E)** The estimated 95% confidence intervals for the priming effect in **(C)**. **(F)** Data used in estimating the priming effect corresponding to **(A,C,E)**. Data adapted by permission from RightsLink Permissions American Psychological Association “Is priming in fragment completion based on a ‘traceless’ memory system?” by Hayman and Tulving (1989), American Psychological Association.

Consider a between-subjects WFC test. A total of 84 students enrolled in a second-year psychology course at the University of Toronto were randomly divided into two groups (one with the last name A-K and the other with the last name L-Z). A set of 48 target English words of intermediate difficulty was selected from a word pool and divided into two wordlists (A and B), with 24 target words in each list. An additional 64 (non-target) English words were used as buffer words. During the study phase, the first group studied wordlist A and the second wordlist B; the wordlist B thus served as non-studied words for the first group, and wordlist A served as non-studied words for the second group. During the test phase, there were two test instructions: the subjects with completion instructions were asked to complete the fragment with any word that comes to mind; subjects with recall instructions were asked to complete the fragment only with studied words. All subjects are randomized into four groups, each to take two tests. Specifically, participants in Group 1 (*N* = 22) conducted two tests under the completion instructions with the same fragment cues during the two tests; participants in Group 2 (*N* = 22) conducted two tests under the completion instructions with different fragment cues during the two tests; participants in Group 3 (*N* = 20) conducted two tests under the recall instructions with the same fragment cues during the two tests; participants in Group 4 (*N* = 20) conducted two tests under the recall instructions with different fragment cues during the two tests. Full data description is available in Experiment 2 in Hayman and Tulving (1989) with study data summarized in Figure 4F.

The potential priming effect of the WFC test is displayed in Figure 4. Using **Eqs.** 2, 4, the *DPE* under completion instruction is 0.104 (*z* = 5.23, *p* = 10^−6^), with a 95% confidence interval (0.066, 0.143); the *DPE* under recall instructions is 0.121 (*z* = 6.36, *p* = 10^−10^), with a 95% confidence interval (0.084, 0.157). The corresponding estimated probabilities (of correct word identification with or without a word study), priming effects and their confidence intervals are shown in Figures 4A,C,E, respectively.

Next, we consider the *DPEs* where the word study primes the identification of a target word in the second test, given the identification of the same target word failed during the first test (see Figures 4B,D). Although the number of the words that failed to be identified during the first test was not reported in Hayman and Tulving (1989) (hence we cannot compute the exact *p*-values), readers could follow the previous example and use **Eq.** 4 in their research when data are available. Nevertheless, we will report the *DPEs* without *p*-values. There are two reasons for this. First, since the total number of the target words studied is large [i.e., 480 words (20 subjects each with 24 target words) and 528 words (22 subjects each with 24 target words) in our case], a positive *DPE* is likely to yield a significance non-zero priming effect. Second, it allows us to numerically compare the priming effects under different experimentation strategies. Specifically, using the same cues in the two tests, the *DPE* under the completion instructions is 0.06; the *DPE* under the recall instructions is 0.048; meanwhile, using the different cues in the two tests, the *DPE* under the completion instructions is 0.122; the *DPE* under the recall instructions is 0.137 (see Figure 4D). The much stronger priming effect observed in both experiments where different cues are provided suggests that given a failed attempt using one cue during the first test, information has potentially been learned by combining the first cue and a *different* cue during the second test.

## Extensions, limitations, future directions, and final remarks

In this article, we defined, quantified, and tested the priming effect using the *potential outcomes framework*. Although we only considered cases involving a binary exposure (having a word study versus not having a word study), the framework can be extended to categorical exposures (e.g., we can code an exposure that does not consist of a word study as *Z* = 0, one that consists of a word study including short words as *Z* = 1, and one that consists of a word study including long words as *Z* = 2). In addition, the framework can be extended to continuous exposures (e.g., when a word study consists of words with different degrees of complexity, we can allow *Z* to take any value between 0 and 100 to indicate complexity of each word). Furthermore, it can be extended to time-dependent exposures [e.g., we can write word studies conducted at different times as *Z*(*t*), for each time *t*]. Finally, it can also be extended to cases where several exposures are concerned (e.g., let *Z*_1_ = reading a list of words, *Z*_2_ = viewing a list of non-word symbols, and *Z*_3_ = listening to a list of words), where the priming effect for each exposure can be estimated when the other exposures are controlled. For example, when estimating the priming effect of symbol recognition (*Z*_2_ = 1 versus *Z*_2_ = 0), one could fix *Z*_1_ and *Z*_3_; namely, the priming effect can be estimated, for example, using *Y*(*X* = *x*, *Z*_1_ = 0, *Z*_2_ = 1, *Z*_3_ = 0) − *Y*(*X* = *x*, *Z*_1_ = 0, *Z*_2_ = 0, *Z*_3_ = 0), where bold *X* indicates all cues used for three studies.

**Eq.** 4 is used to test the significance of the priming effect with binary exposures. When the exposures are multivariate, continuous, or time-dependent, the test can be carried out by first arranging the exposure and outcome as explanatory and dependent variables in a regression setting, and then testing the exposure effect by examining the significance of the (regression) parameters. For example, when there are three types of word studies (no word study, a word study with short words, and a word study with long words), one can consider a regression model with a block design, where each block consists of subjects from one of the three groups. The estimated regression parameter for the block variable then indicates the priming effect between two paired groups. When the exposure is time-dependent^17^, one could refer to functional regression models, wherein *Z*(*t*) and *Y*(*t*) are treated as functional regressors and responses, respectively (Ramsay and Silverman, 1997).

The proposed method aimed at providing a framework that could estimate and validate analytically priming in between-subjects designs. It nonetheless has a few limitations. First, we demonstrated the method using data from previous experiments (that are not primarily intended to evaluate the priming effect but to assess the independence of successive tests). Inevitably, this restricted our arguments; future research may verify and expand our analysis to general priming research. Future studies may also extend to cases with a larger sample, and non-twin studies need to examine covariant control under the potential outcomes framework (see section “Estimating Priming Effects With Covariates”) and its utility on providing an estimated priming effect that is less biased. In parallel, future research may further consider twin studies where the subjects are nearly perfectly matched. Second, the method we introduced rests on the Neyman–Rubin potential outcomes framework. There is, however, on the one hand, not as of yet a consensus that one causal framework is better than others, although we have discussed the advantages of the Neyman–Rubin framework in estimating potential priming effect (especially its mathematical representations). On the other hand, we recognize that despite differences and disagreements [e.g., see Gelman’s blog post (Gelman, 2009) and discussion under the post], there is some commonality between Neyman–Rubin’s and Pearl’s frameworks (Section 7.4.5 of Pearl, 2009b), and there exists “a happy symbiosis between graphs and counterfactual notation” (Section 7.4.4 of Pearl, 2009b). In this study, while we present the arguments using the Neyman–Rubin model, we have adopted Pearl’s diagram representation (although without graphic notations) to illustrate the experiments. We do so without implying that one framework is superior to the other. Future studies may theoretically compare the Neyman–Rubin approach with Pearl’s approach in detail for studying potential causal priming (e.g., their mathematical or empirical equivalence or difference). Further research may also incorporate Campbell’s approach to identify potential threats that may impair the validity of inferences made on the estimated priming effect.

Sometimes, investigators studying the priming effect may observe post-treatment variables (i.e., variables obtained after the word study *Z* is assigned). Examples of post-treatment variables are (a) a measure of subjects’ compliance to the originally assigned word study – a subject chooses not to take the word study after it is assigned; (b) in studies with a long time interval between two priming tests, whether or not the subject drops out is a post-treatment variable (missingness of outcome); (c) in longitudinal (priming) studies involving patients with severe amnesia, the outcome can be censored (i.e., not recorded due to death); (d) in studies investigating priming effects for patients with brain disorders, surrogate variables of disease progression and fluctuation, such as the degree of memory loss, are post-treatment variables. The estimators provided in this article can only be used to adjust for pre-treatment variables; if one adjusts for post-treatment directly using the framework outlined in this article, the estimated effects are no longer causal (Frangakis and Rubin, 2002).

It is worthwhile noting that besides the potential outcome framework (by comparing outcomes on randomly selected or matched subjects, or subjects with covariates adjusted), priming can also be estimated using a repeated-measures design, in which *all* subjects are exposed first to half of the target words and then another half of the target words (e.g., see Challis and Brodbeck, 1992). The priming effect can then be estimated as the difference between the proportion of fragments of studied words completed and the proportion of fragments of non-studied words completed for the same subject (and averaged across all subjects). Instead of matching two groups of subjects as proposed in this study, the key to using the repeated-measures designs is to match the length, frequency, etc., of the words and randomize the words employed. Whereas this indeed provides an alternative (and potentially convenient^18^) approach to assess the priming effect, and we welcome future research to compare this approach with the potential outcomes framework; a key concern with this method is the carryover or learning effect. The carryover or learning effect here is not necessarily the phenomenon where after studying the same (or similar) words multiple times, the earlier word study and identification may improve the same subject’s later word identification; rather, it also includes the phenomenon where the experiment mechanism of the first repeated-measures study may improve learning during the second repeated-measures study. We have seen such a carryover or learning effect during a smartphone-based cognitive test, where even though different tests (e.g., drawing different shapes) were given to the same subjects over time, their performance improved. For the WFC test, it may be possible that the subjects learned some rules (despite not being informed) during the first half of the experiment or became more focused during the second half either because they had guessed the approximate rule or because they had realized that the word study may be an important part (since, for example, two wordlists had been given sequentially) to their performance of the experiment. Future studies could examine the existence of such a learning or carryover effect, and if exists, whether and how it would affect estimating the priming effect.

There are times where even randomization becomes impossible. For example, suppose one is interested in studying how a new medicine affects priming; in this case, we have two potential causes: a word study (*Z*) and medication (*Med*). It is unethical to assign a group of 45-year-old healthy subjects to take a new drug to investigate whether the drug improves priming at 50. In addition, there is likely another source, say, the socioeconomic status (which may be related to the affordability of new drugs) or genetics (if there is a family history of memory problems, one may be more willing to take the drug), that may be associated with taking the drug and/or developing memory problems at 50. Similarly, it would be difficult to estimate the effect of taking the drug on improving priming by comparing the performance of an individual at 50 who had taken the drug with his or her performance at 50 had he or she not taken the drug. To solve these issues, the propensity score matching (PSM) estimates the treatment effect by comparing the outcomes of the subjects under treatment (e.g., taking the drug) with a set of “matched” subjects without treatment (e.g., having not taken the drug) (Rosenbaum and Rubin, 1983; Dehejia and Wahba, 1999, 2002; Caliendo and Kopeinig, 2008). More concretely, one could first compute the propensity score of A’s and B’s taking the drug based on their gender, economic, social, genetic, and demographic backgrounds, and choose two individuals C and D from a group of 50-year-olds who had not taken the drug but have propensity scores (of taking the drug during their younger years) closest to A’s and B’s, respectively. Subsequently, A and C will receive a word study, and B and D will not. Following the previous notations, we have *Y*_*A*_ (*X*_*j*_, *Z* = 1, *Med* = 1), *Y*_*B*_(*X*_*j*_, *Z* = 0, *Med* = 1), *Y*_*C*_(*X*_*j*_, *Z* = 1, *Med* = 0), and *Y*_*D*_(*X*_*j*_, *Z* = 0, *Med* = 0), for 1 ≤ *j* ≤ *M*, where *M* target words are considered. Then, the priming effects for the group taking the drug and the one not taking the drug are $DPE_{Med=1}=\frac{1}{M}\left\{\sum_{j=1}^{M}Y_{A}\left(X_{j},Z=1,Med=1\right)-\sum_{j=1}^{M}Y_{B}\left(X_{j},Z=0,Med=1\right)\right\}$ and $DPE_{Med=0}=\frac{1}{M}\left\{\sum_{j=1}^{M}Y_{C}\left(X_{j},Z=1,Med=0\right)-\sum_{j=1}^{M}Y_{D}\left(X_{j},Z=0,Med=0\right)\right\}$, respectively. Subsequently, we can estimate the drug effect on priming using *DPE*_*Med* = 1_ − *DPE*_*Med* = 0_. Note that for simplicity, only one individual is considered for each of the 2 × 2 factors; one can relatively easily extend the above to include multiple subjects in each group.

Although we have throughout focused on a type of non-semantic priming, other studies have reported that new semantic knowledge can be acquired among (even) patients with amnesia. For example, the learning of specified target words in meaningful texts, statements of facts about people and places, specified target words as parts of meaningful sentences, new computer-related vocabulary, computer commands, semantic interpretations of ambiguous descriptions of situations and events, and production of words to cues consisting of the initial letters of words (see Hayman et al., 1993 for a summary of studies). Future studies should independently verify the extent to which the framework introduced in this article can be used to estimate causal semantic priming. A beginning can, perhaps, be made by reporting the individual ratings of the meaningfulness of the target words (e.g., during a word study, every participant is to rate on a scale of 0–10, the meaningless of each studied word), and subsequently treating the ratings as covariates.

In conclusion, we define, quantify, and test the causal priming effect using the *potential outcomes* framework. Applying data from a between-subjects word completion test, we demonstrate that the framework identifies a significant priming effect from a word study to cue-based word identification, under both completion and recall instructions; the priming effect under the recall instructions is more significant than that under the completion instructions. Furthermore, when there are two consecutive tests, the framework shows that even if the word identification failed during the first test, there is likely a priming effect from the initial word study to the second word identification, regardless of the type of instructions and whether the same or different cues are used in the two tests. In addition, there is a stronger priming effect in experiments where different cues are provided, suggesting that given a failed attempt using one cue during the first test, additional information may have been learned by combining the first cue and a different cue during the second test. Finally, our explorations show that what has been intuitively used by scholars to estimate the priming effect in the past has a meaningful mathematical basis.

## Data availability statement

Publicly available datasets were analyzed in this study. These data can be found here: Hayman and Tulving (1989).

## Ethics statement

The studies involving human participants were reviewed and approved by the University of Toronto. Written informed consent for participation was not required for this study in accordance with the national legislation and the institutional requirements.

## Author contributions

OC designed the potential causal priming framework (based on Neyman and Rubin’s works on causal inference and Tulving and Schacter’s works on priming) and performed the analysis. HP provided computational support. HC and GN provided neurobiological and psychological interpretations. TQ provided statistical support. MV provided funding, support, and guidance. OC wrote the manuscript, with comments from all other authors. All authors contributed to the article and approved the submitted version.

## Footnotes

1 Either viewing the target words or not viewing them; one cannot un-view the words that one had viewed.

2 Here, *do*(*X* = *x*) means the model forces *X* to take the value of *x*; in other words, one sets *X* (*via* intervention) to be a constant value *x*.

3 Consider two experiments: *A X O_1_ vs*. *A* [ ] *O*_2_, where *A, X* (or lack thereof), and O are ordered from left to right in time, and *A, X*, and *O* indicate a random sampling assignment, a treatment, and the outcome, respectively. In Campbell’s approach, the presence of *X* on the left of *O*_1_ means *O*_1_ is the outcome of a group after receiving a treatment *X*, and the absence of *X* (*i.e.*, blank space) on the left of *O*_2_ means *O*_2_ is the outcome of another group without receiving a treatment.

4 The letter *A* in Campbell’s approach is equivalent to the randomization and matching mechanism in Neyman-Rubin’s approach, or in Campbell’s words: “*A* is the point of selection, the point of allocation of individuals to groups … At time *A* the groups were equal, even if not measured…”

5 Pearl’s circles and arrows are equivalent, in spirit, to Campbell’s letters and orders: in Campbell’s notation, if *X* is placed on the left of *O*, it implies there is a directed arrow from *X* to *O*.

6 Namely, *P*{*Y*|*do*(*Z*) = *z*} = *P*{*Y*(*z*)}, where *P*{*Y*|*do*(*Z*) = *z*} (in Pearl’s language) means forcing *Z* to take the value *z* by removing all father nodes of *Z*, and *P*{*Y*(*z*)} (in Neyman-Rubin’s language) means the potential outcome of Y under *z*.

7 Suppose (1) U and W both cause X, (2) U and W cause T and Y, respectively, and (3) T causes Y. Using Pearl’s method, the relationship from T to Y is causal. But using Neyman-Rubin’s method, by controlling for X, the relationship from T to Y is not causal.

8 We attribute a part of the summary between Neyman-Rubin’s and Pearl’s methods to works from Peng Ding.

9 The discussion was on primates.

10 This indicates the study phase did not affect the WFC.

11 The definition of “match” here is more restricted than it is in the context of propensity score matching, as we consider the matched pair to have identical potential outcomes. We use this term for illustration of causal effect rather than estimation.

12 We need both potential outcomes to be equal; not just the potential outcome under treatment (*i.e., Z* = 1, namely had a word study been conducted).

13 Random variables related to individuals are assumed to be independent and identically distributed (*i.i.d.*). Thus, we can write the indicator function *I*_Y*_i_*_(X = x, Z = z) = 1 as *I*_Y_(X = x, Z = z) = 1.

14 It is natural to assume *C* = *D*; otherwise, we can replace “improvement” with “the difference.”

15 Readers can relatively easily extend it to more complicated cases involving unbalanced groups, where the numbers of subjects and/or target words in two groups are unequal.

16 Statistically speaking, adjusting for covariates in a randomized study improves the precision (reduces the variance) of the treatment effect estimator (see Moore et al., 2011; Qian et al., 2018).

17 For example, at each time *t*, a study *Z*(*t*) is assigned to individuals, and a word identification *Y*(*t*) is observed. This is particularly useful when the sample consists of subjects whose implicit memory degenerates over time.

18 For example, when there are no matched samples (but see Propensity Score Matching (PSM) and covariates adjustment discussed in this paper), and that the carryover or learning effect is ignorable, a repeated-measures design is attractive.
